# Personalizing Transient Noise Reduction Algorithm Settings for Cochlear Implant Users

**DOI:** 10.1097/AUD.0000000000001048

**Published:** 2021-04-08

**Authors:** H. Christiaan Stronks, Annemijn L. Tops, Phillipp Hehrmann, Jeroen J. Briaire, Johan H. M. Frijns

**Affiliations:** 1 Department of Otorhinolaryngology, Leiden University Medical Center, Leiden, The Netherlands; 2 Advanced Bionics, LLC, Valencia, California, USA; 3 Leiden Institute for Brain and Cognition, Leiden University, Leiden, The Netherlands.

**Keywords:** Cochlear implants, Front-end processing, Impulse noise, Noise, Sensorineural hearing loss, Speech intelligibility

## Abstract

**Objectives:**

Speech understanding in noise is difficult for patients with a cochlear implant. One common and disruptive type of noise is transient noise. We have tested transient noise reduction (TNR) algorithms in cochlear implant users to investigate the merits of personalizing the noise reduction settings based on a subject’s own preference.

**Design:**

The effect of personalizing two parameters of a broadband and a multiband TNR algorithm (TNR_bb_ and TNR_mb_, respectively) on speech recognition was tested in a group of 15 unilaterally implanted subjects in cafeteria noise. The noise consisted of a combination of clattering dishes and babble noise. Each participant could individually vary two parameters, namely the scaling factor of the attenuation and the release time (τ). The parameter τ represents the duration of the attenuation applied after a transient is detected. As a reference, the current clinical standard TNR “SoundRelax” from Advanced Bionics was tested (TNR_bb-std_). Effectiveness of the algorithms on speech recognition was evaluated adaptively by determining the speech reception threshold (SRT). Possible subjective benefits of the algorithms were assessed using a rating task at a fixed signal-to-noise ratio (SNR) of SRT + 3 dB. Rating was performed on four items, namely speech intelligibility, speech naturalness, listening effort, and annoyance of the noise. Word correct scores were determined at these fixed speech levels as well.

**Results:**

The personalized TNR_mb_ improved the SRT statistically significantly with 1.3 dB, while the personalized TNR_bb_ degraded it significantly by 1.7 dB. For TNR_mb_, we attempted to further optimize its settings by determining a group-based setting, leaving out those subjects that did not experience a benefit from it. Using these group-based settings, however, TNR_mb_ did not have a significant effect on the SRT any longer. TNR_bb-std_ did not affect speech recognition significantly. No significant effects on subjective ratings were found for any of the items investigated. In addition, at a constant speech level of SRT + 3 dB, no effect of any of the algorithms was found on word correct scores, including TNR_mb_ with personalized settings.

**Conclusions:**

Our study results indicate that personalizing noise reduction settings of a multiband TNR algorithm can significantly improve speech intelligibility in transient noise, but only under challenging listening conditions around the SRT. At more favorable SNRs (SRT + 3 dB), this benefit was lost. We hypothesize that TNR_mb_ was beneficial at lower SNRs, because of more effective artifact detection under those conditions. Group-averaged settings of the multiband algorithm did not significantly affect speech recognition. TNR_bb_ decreased speech recognition significantly using personalized parameter settings. Rating scores were not significantly affected by the algorithms under any condition tested. The currently available TNR algorithm for Advanced Bionics systems (SoundRelax) is a broadband filter that does not support personalization of its settings. Future iterations of this algorithm might benefit from upgrading it to a multiband variant with the option to personalize its parameter settings.

## INTRODUCTION

Cochlear implant (CI) users can generally understand speech well in quiet. In background noise, however, the speech understanding of listeners with a CI declines steeply (Nelson et al. 2003; Zeng et al. 2005). Background noise can consist of sounds varying in intensity and duration. Transient noise is defined as disrupting sounds with a fast onset, a fast decay and a maximum duration of 1 second (Henderson & Hamernik 1986; Hernandez et al. 2006). Most research has focused on stationary noise and relatively slowly fluctuating noise such as competing talkers and babble. However, transient noise is actually surprisingly common, making up approximately one third of environmental sounds. Transient noise has been reported to be experienced as moderately annoying, on par with stationary noise (Hernandez et al. 2006).

**Fig. 1.**
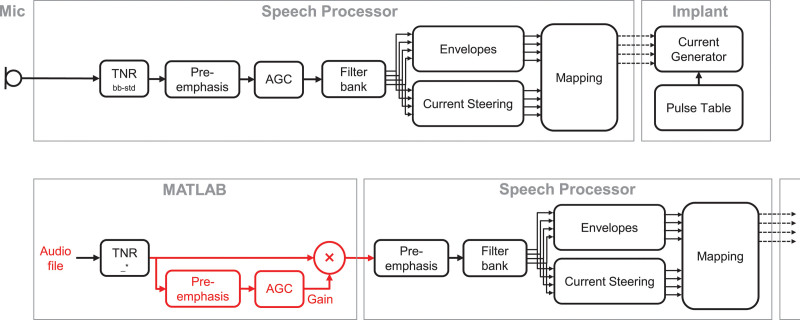
Block diagrams of the sound coding in the HiRes Fidelity 120 strategy. Clinical strategy (A) and experimental sound processing used in this study (B). For experimental purposes, transient noise reduction (TNR) algorithms and the broadband dual-loop adaptive gain control (AGC) were simulated in MATLAB (red blocks). The implant block is omitted in (B). Red boxes in (B) reflect differences between the clinical (TNR_bb-std_) and experimental sound coding strategies (TNR___*). See Text for further details. TNR_bb-std_ indicates clinical standard broadband transient noise reduction algorithm SoundRelax.

In response to these findings, Phonak developed the transient noise reduction (TNR) algorithm SoundRelax for use in hearing aids (HAs), which has been available since 2006 (Dyballa et al. 2015). It is currently also available in CIs from Advanced Bionics and will be referred to here as the clinical standard, broadband TNR (TNR_bb-std_). The algorithm has been described in detail by Dyballa et al. (2015, 2016). It is a front-end processing strategy that is applied before the broadband dual-loop adaptive gain control (AGC) in the Advanced Bionics HiRes Fidelity 120 speech processing chain (Fig. 1A). TNR_bb-std_ continuously monitors the slope and amplitude of the full broadband envelope of the incoming signal in near real-time. When these parameters exceed a certain threshold, the broadband signal to the AGC is shortly attenuated. The dual-loop AGC has a slow- and fast-acting detector that can also effectively reduce transients (Moore & Glasberg 1988; Boyle et al. 2009). The slow detector is designed to adjust overall sound level gradually to adapt to different listening situations. Its compression ratios can be high, but it is nonetheless unsuitable for transient detection because the gain is changed too slowly. The fast-acting detector has a more moderate compression ratio but is theoretically able to detect and suppress transients (Boyle et al. 2009). However, even fast-acting AGCs are typically not very suitable for suppressing transients, as their attack time is mostly in the range of 3 to 5 ms, which is too slow to suppress the rapid onset of many transients. TNR_bb-std_ was specifically designed to suppress transients with onset times in the order of 1 ms. It features a fast attack time and a high compression ratio to suppress high-intensity transients (Dingemanse et al. 2018).

Studies on the effectivity of TNR_bb-std_ and related algorithms in CI users and listeners with HAs have shown mixed results. Most of them revealed a significant benefit on one or more subjective rating scales, for example, annoyance level of the transient noise, or overall sound comfort (Keidser et al. 2007; Liu et al. 2012; Dingemanse et al. 2018; Keshavarzi et al. 2018), but failed to show a significant effect on speech intelligibility (Keidser et al. 2007; Dingemanse et al. 2018). DiGiovanni et al. (2011) reported no significant effects on any of the subjective ratings tested, but they did find a significant, yet marginal improvement of 4.4% of the speech intelligibility across various noise conditions.

The variability of the benefits of TNR algorithms between studies may have been caused by a multitude of factors, such as the specific TNR algorithm used, the type and sound level of the noise and speech material, the target population (HA or CI users), and the outcome measure and type of test used. However, even studies with very similar designs have produced contradictory results. For instance, annoyance ratings of door slams by Siemens HA users were significantly reduced by a TNR algorithm. Door slams are characterized by steep rise and fall times. The same TNR algorithm applied to slowly modulated babble noise did not result in significant effects on annoyance ratings (Keidser et al. 2007). By contrast, in a similar study that tested the same TNR algorithm, Digiovanni et al. (2011) showed a significant decrease in annoyance when multi-talker babble was rated, but not when door slams were used as noise. Noise and speech levels were in a similar range between the studies, but different speech tests and rating scales were used.

A convincing benefit of transient-noise suppression on both subjective rating measures and speech intelligibility was found in a study of Dyballa et al. (2016), where a novel multiband variant of TNR_bb-std_ was designed (TNR_mb-std_). Because the overall long-term spectrum of transient noise covers a wider frequency range than speech, detection and suppression of transients may be more efficient in frequency bands outside the speech spectrum. TNR_mb_ divides the input audio signal into four frequency bands (0 to 1000, 1000 to 2000, 2000 to 4000, and >4000) and independently applies the detection and attenuation to these bands using the same detector as TNR_bb-std_. The attenuations are proportional to the band-specific transient amplitudes and vary from 10 to 30 dB (Dyballa et al. 2016). The authors reported significantly better speech intelligibility with TNR_mb-std_ in transient noise than with the TNR switched off (TNR_off_), and it significantly outperformed TNR_bb-std_. Perceived comfort and speech clarity were significantly higher for TNR_mb-std_ than for TNR_off_, or TNR_bb-std_.

Dyballa et al. (2015) made a step toward the evaluation of different settings of the noise reduction strength, by comparing two variants of a broadband TNR algorithm in CI users. One was designed to aggressively attenuate transients (TNR_bb-high_), while the other had a more moderate attenuation (TNR_bb-low_). Both TNR variants resulted in significant benefits on speech intelligibility and subjective preference ratings, but no significant differences between the two algorithms were shown.

The aim of our study was to investigate the merits of personalizing the attenuation and τ of two different TNR algorithms in an attempt to deliver personalized care for individual CI users. Personalization of TNR algorithms may hold promise, because the tolerance of CI users to noise transients varies substantially (Dingemanse et al. 2018). One possible reason for this observation is that above most comfortable stimulation (M) levels, the perceived loudness growth becomes unpredictable (Fredelake et al. 2014). M levels can be determined for each individual electrode by the audiologist by slowly increasing the stimulation level up until the point that the associated loudness percept is most comfortable. The loudness growth above M levels is limited by the AGC, which compresses the acoustic dynamic range above M levels. M levels differ between electrodes and vary from person to person. Because of the variability of M levels and the unpredictable loudness growth above M levels, loud sounds will be perceived differently from person to person. This may well be the underlying reason of the variable tolerance to high-intensity noise transients seen in CI users (Dingemanse et al. 2018). A CI user with a low tolerance may prefer more aggressive filtering than others with a higher tolerance threshold. On the other hand, every noise reduction algorithm inevitably distorts the (speech) signal as well, and the tolerance to speech distortion also differs between CI users (Kokkinakis et al. 2011). For users that tolerate little speech distortion, it is conceivable they prefer less aggressive attenuation to limit distortion of the speech signal.

The specialized fitting methods in terms of M and threshold (T) levels for CIs are very different from those applied in HAs, and therefore dedicated studies to optimize the fitting of TNR algorithms in CI users are warranted. In addition, CI users may benefit less from TNR algorithms. CI users generally experience difficulty understanding speech even in positive signal-to-noise ratios (SNRs), while HA users perform better and can tolerate even negative SNRs. Given that TNRs expectedly more effectively detect and suppress transients at low SNRs (Dyballa et al. 2016), they may be less beneficial for CI users than for people with a HA.

Two important parameters that are available to personalize and optimize TNR_bb_ and TNR_mb_ are the amount of attenuation of the noise transient and the release time τ. The attenuation parameter determines the amount of suppression applied after a transient is detected, and τ governs how long the attenuation is applied for after a transient is detected. Larger attenuation and longer release times improve suppression of noise transients but also increasingly distort the speech signal.

In this study, we tested a variant of TNR_bb-std_ and TNR_mb_ where both the attenuation and τ could be varied (TNR_bb_ and TNR_mb_, respectively). CI users were presented speech-in-noise, and they were provided with a graphical user interface (GUI) that allowed them to manually vary attenuation and τ in real-time to find their preferred combination for optimal speech intelligibility and/or listening comfort. The potential benefit of personalizing attenuation and τ has not yet been investigated before in CIs. These personalized algorithms were tested against TNR_off_ and against the current clinical standard TNR_bb-std_.

## MATERIALS AND METHODS

### Study Design and Participants

This was a study with a single-masked (the experimenter was always aware of the condition being tested, the subject was not), cross-over design (each subject was their own control). To prevent bias, the experimenters did not share information with the subjects about the study design or on the order of tests. The subjects were only told that different settings were being tested of a transient-noise suppression algorithm using a speech-in-dish-clatter test. The study consisted of four test sessions that were scheduled on different days, each session lasting approximately 2 to 3 hours. In the first two, speech-in-noise testing was performed to test the effectiveness of personalized settings for TNR_bb_ and TNR_mb_, and to compare them against TNR_off_ and TNR_bb-std_. The first session was used to obtain the test, the second provided a retest. The four conditions and test lists were randomized and the patients were unaware which of the conditions was being tested. In a 3rd session, the optimized group-based settings were tested of TNR_mb_. In the 4th session, subjective rating experiments were done.

Eighteen CI users were initially included in this study. All but one subject were unilaterally implanted subjects. The one bilateral CI user was tested using one ear only, namely the one that was implanted first, that is, the better hearing side. One subject decided to exit the study during the first session (S02). Two subjects (S08, S13) were identified as outliers, because they had disproportionately large speech reception thresholds (SRTs), corresponding to speech levels exceeding 90 dB SPL equivalent. Because such high speech levels are not representative for everyday life, the data from these patients were excluded from the study. The demographics of the remaining 15 participants (4 males, 11 females; 21 to 79 years old; median 64 years old) are shown in Table 1. They were implanted with a HiFocus Mid-Scala or 1j electrode array (Advanced Bionics, LLC, Valencia, CA, USA) at least 16 months before testing (mean 5.31 years) and they had free-field consonant-vowel-consonant (CVC) phoneme scores at 65 dB SPL in quiet of at least 82% (mean 91%). Eighty-two percent corresponds approximately to the median score for this type of test in our center, that is, this group of subjects was better than average overall.

**TABLE 1.** Subject demographics

Subject ID	Sex	Age (yr)	Etiology	CVC score (%)	CI experience (yr)	CI device	Contralateral ear	Contra Fletcher index
1	F	62	Progressive	85	3	MS	HA	117
3	F	68	Genetic	86	4	MS	HA	95
4	M	70	Otosclerosis	92	5	MS	HA	102
5	F	50	Meningitis	82	3	1J	NH	15
6	F	65	Progressive	95	2	MS	HA	68.3
7	F	21	Congenital?	96	1	MS	HA	85
9	M	68	Genetic	90	5	MS	—	91.7
10	F	74	Sudden deafness	86	4	MS	HA	108
11	F	52	Meningitis	98	16	CII	CI	N/A
12	F	62	Genetic	93	7	1J	HA	103.3
14	F	64	Genetic	89	4	MS	—	72
15	M	78	Progressive	86	7	1J	HA	95
16	F	63	Genetic	96	4	1J	HA	67
17	M	61	Otosclerosis	95	4	MS	—	100
18	F	79	Progressive	83	3	MS	HA	118

90K/1J, HiRes 90K HiFocus 1j; 90K/MS, HiRes 90K HiFocus Mid-Scala; CI, cochlear implant; CII, CII HiFocus II with positioner; CVC, consonant-vowel-consonant; F, female; HA, hearing aid; ID, identification number; M, male; NH, normal hearing.

All participants were Dutch native speakers, except for one person from the United Kingdom. This person had been living in the Netherlands for the last 40 years, was sufficiently proficient in the Dutch language, and met the study inclusion criteria and was hence included in this study. The participants were fitted with an Advanced Bionics Harmony processor with their own threshold and maximum comfort levels. Any additional assistive hearing devices (HAs, contralateral routing of signals devices) were removed before experimentation, and no other noise reduction algorithms were applied (e.g., SoftVoice or ClearVoice).

This study adhered to the tenets of the Declaration of Helsinki (World Medical Association 2013) and the study protocol was approved by the Institutional Review Board of the Leiden University Medical Center. All participants provided written informed consent.

### Hardware and Software Setup

TNR_bb-std_ is available on Advanced Bionics speech processors. However, to facilitate the flexible adjustment of the attenuation and τ in the TNR_bb_ and TNR_mb_ algorithms, the speech processing was partly taken over by a laptop using a MATLAB programming environment (R2017b; MathWorks, Inc., Natick, MA, USA), as shown in Figure 1. In the normal HiRes F120 coding strategy, the microphone input is fed through the TNR algorithm after which it is sent to the preemphasis and AGC, respectively (Fig. 1A). In this study, the microphone input was substituted by audio files. The TNR algorithms, preemphasis, and the broadband, dual-loop AGC were simulated in MATLAB to allow for precise control of the attenuation and τ (Fig. 1B). After the digital audio files were fed through the simulation, the signal was sent from the laptop to a Harmony speech processor using DirectConnect (Fig. 1B). Because the AGC was emulated in MATLAB, it was disabled in the speech processor using the clinical fitting software (SoundWave), which sets it to linear gain. However, disabling of the preemphasis in the speech processor was not possible in this manner. To simulate the processing chain correctly, the ACG gain needed to be applied to the audio input to the speech processor, but without the preemphasis, as it was applied by the speech processor. To achieve this, a bypass was designed in MATLAB (indicated by the red boxes in Fig. 1B) that computed the AGC gain based on the signal including preemphasis, but applied it to the simulated TNR output without preemphasis (box labeled with “X” in Fig. 1B). After the preemphasis step on the speech processor, all the components present in the clinical speech coding strategy were also applied to the experimental signal.

The auditory stimuli were stored on hard disc and processed on an external USB sound card (RME Babyface USB 2.0 Audio Interface, 192 kHz DA conversion; Audio AG, Haimhausen, Germany) and sent to the auxiliary input of a Harmony processor (Advanced Bionics LLC, Valencia, CA, USA) using the DirectConnect hook (Firszt et al. 2009). The setup was calibrated such that the electric output was equivalent to the acoustic sound pressure level when processed through the microphones of the speech processor. The volume setting of the processor was set at 100% by default but adjusted upward in some subjects who reported the sound levels to be too low.

### Speech and Noise Stimuli

The speech material consisted of the Flemish/Dutch matrix sentences (Luts et al. Reference Note 1). The primary outcome parameter was the SRT in noise, that is, the SNR where the subject could identify 50% of the words (Brand & Kollmeier 2002). The noise type used was a “cafeteria” noise, consisting of a mix of clattering dishes, and reverberant multi-talker babble. The noise was identical to the one described previously (Dyballa et al. 2016). To the normal-hearing ear, the noise was acoustically perceived as a loud clatter with a damped murmur in the background. In line with that previous study, the cafeteria noise was presented at a constant root-mean-squared (rms) level of 80 dB SPL. The noise started 1.5 seconds before the speech onset to allow the AGC of the speech processor to set in.

### Personalizing the TNR Algorithms

**Fig. 2.**
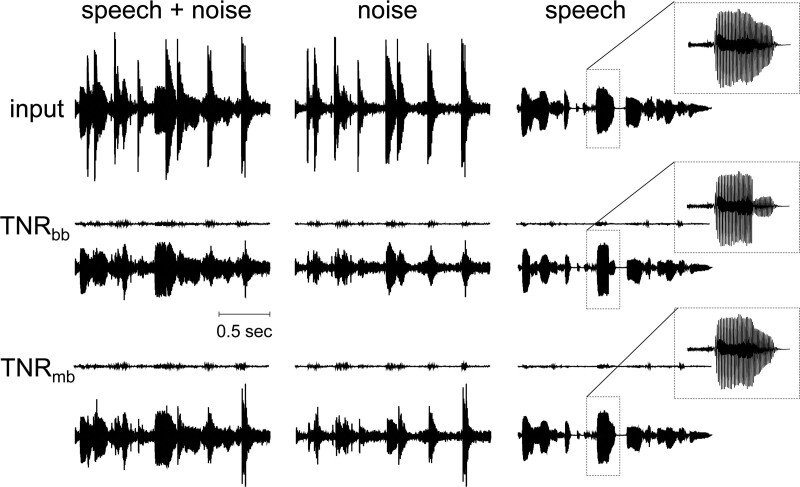
Sample input and output waveforms of speech and noise before and after filtering with a broadband transient noise reduction (TNR_bb_) algorithm and a multiband variant (TNR_mb_). Speech was a sentence of the Matrix test. Noise was cafeteria noise, including dish clatter and babble noise. The visible impulse noise is predominantly the dish clattering. The low-amplitude traces shown for TNR_bb_ and TNR_mb_ are plotted on the same scale as the input. The traces below them are scaled up by 12 dB. Settings were default, that is, scaling factors of attenuation and τ equal to 1, such that TNR_bb_ equaled the standard clinically available filter (TNR_bb-std_).

TNR_bb_ was the same algorithm as the current clinical standard SoundRelax (TNR_bb-std_) where the attenuation and τ could be varied and hence personalized to the subject’s preferred settings. TNR_mb_ was a similar algorithm with variable attenuation and τ, but it applied detection and attenuation in four separate frequency bands (0 to 1000, 1000 to 2000, 2000 to 4000, and >4000 Hz) (Dyballa et al. 2016). Personalization was realized at the start of the first session by presenting the subjects with speech in cafeteria noise and a GUI that ran in a MATLAB environment. The GUI consisted of a field on a touchscreen where the axes represented the parameters attenuation and τ. A red circle indicating the current setting could be dragged anywhere across the field. Both parameters were then adjusted in real-time, allowing the subject to apply a scaling factor ranging from 0 to 2 to the attenuation and τ, relative to the standard settings of TNR_bb-std_ (30 dB and 30 ms, respectively). Hence, attenuation could be varied from 0 to 60 dB, and τ from 0 to 60 ms for TNR_bb-std_. For TNR_mb_, each frequency band had a slightly different τ ranging from 30 to 40 ms by default. These values were based on informal pilot experiments performed at Advanced Bionics’ European Research Center (Hannover, Germany). The scaling factor also ranged from 0 to 2 for this algorithm and was applied to the default τ value of each band. Hence, τ settings could be varied between 0 and 60–80 ms, while the scaling of the attenuation was identical to TNR_bb_. The personalized settings were compared against the clinical standard TNR_bb-std_ and TNR_off_. Sample input and output speech and noise waveforms are shown in Figure 2 under default conditions (scaling factors for attenuation and τ equal to 1). Because default settings of attenuation and τ were used, TNR_bb_ equals TNR_bb-std_. Marked effects on the amplitude of the dish clatter can be seen for both algorithms in the figure.

Subjects were asked to choose that setting where they found the speech intelligibility to be best. This task was performed in three iterations per setting. To increase the accuracy of the estimated preferred setting, the available parameter range of the scaling factors of τ and attenuation was halved each iteration. Each new range was centered on the previously selected value, and constrained to the original limits of the first iterations. Every time the subject picked a setting, the starting position of the indicator changed, forcing the subjects to readjust it again. For each of these iterations, the subjects could take as much time as they liked. We did not consistently register whether subjects were able to recognize the speech during the personalization phase for TNR_bb_ and TNR_mb_. However, from at least five subjects, we know they were not able to discern any speech during the determination of individual preference. The reason for this was that the SNR was fixed to a relatively low value of –2 dB. This value was chosen based on the average SRT obtained in a group of CI users that were tested with the German matrix test. In that study, the same cafeteria noise and sound level (80 dB SPL) were used as in the present study (Dyballa et al. 2016). The fixed speech level in that study was based on the average SRT (72 dB SPL) increased by 6 dB (to 78 dB SPL), yielding an SNR of –2 dB that was sufficiently favorable to allow their subjects to recognize the speech. Apparently, the same SNR proved to be too low for a subgroup of our subjects. They were instructed to select the most comfortable settings instead, that is, where the speech was best noticeable, or where the noise was the least annoying. The preferred attenuation and τ settings were determined once at the start of the 1st session and used again in the following sessions.

### Adaptive Testing of Speech Recognition

Speech recognition was assessed with the Dutch/Flemish Matrix test using custom-built software in a MATLAB R2017b programming environment. The speech corpus of the Matrix test consisted of sentences of five words. Each sentence had the same fixed grammatical syntax, namely: name—verb—numeral—color—object, for example, “David has five white cloths” (Luts et al. Reference Note 1). The outcome measure was the SRT, that is, the SNR required to obtain a word score of 50% correct. Each run consisted of 20 sentences. After each presentation, the speech level was adaptively varied using the up-down method (Levitt 1971). The step size was decreased after each reversal to a minimum of 0.1 dB. The reduction of the step size was managed adaptively and depended on both the step size and the word correct score of the previous trial. Typically, the speech level varied several dB in the first few trials of the run, while in the later trials, when the speech level converged onto the SRT, the variation was not more than 0.2 dBA. To determine the SRT, the speech levels of the last eight trials (including the level that would have been played on a 21st trial, given the result of the final 20th trial) were averaged to obtain the final SRT. Subjects verbally repeated the words that were recognized, if any. Guessing was allowed. The scoring was performed manually by the experimenter. No feedback was given to the subject either during, or after the run, except during the practice runs when necessary. Two practice runs were performed at the beginning of each test session to reduce learning effects, as suggested by (Kollmeier et al. 2015). Most participants (12\15) had prior experience with the Matrix test. The first practice run was performed in quiet, the other in noise. The SRT obtained in noise was used as the starting SNR of the subsequent tests.

### Subjective Ratings

The different TNR algorithms were tested on a subset of the subjects (n = 11) at a constant SNR (SRT + 3 dB) to obtain subjective ratings on a Likert scale (0 to 10). Four of the 15 subjects included in the first three sessions were excluded from this last test session, because of time constraints. At the start of this last session, the subjects performed two practice runs. Then, the SRT was determined (as in sessions 1 to 3, again using the cafeteria noise) without any TNR algorithm. The ratings were subsequently performed based on a set of ten sentences, presented at a constant speech level of SRT + 3 dB. The same noise level was used as in the first three sessions (80 dB SPL equivalent). Subjects were asked to rate the following items: speech intelligibility, speech naturalness, listening effort, experienced annoyance of the noise, and overall experience of the speech and noise. Ratings were labeled from 0 (minimal), 5 (moderate) to 10 (very). For instance, for speech recognition, the question was posed as “How well was the speech recognizable?” and the labels would read as follows: 0: “not at all,” 5: “moderately well,” and 9: “very well.” Overall experience was not labeled, as the educational grading system in the Netherlands typically goes from 0 (lowest) to 10.

The first experimental run was always done without noise, which served as an anchor to the most favorable testing condition. For the first two subjects tested, the following runs were all randomized. For the subsequently tested nine subjects, however, we anchored the ratings further by presenting a second anchor after the first, namely speech in noise without any TNR algorithm, which expectedly yielded the worst SNR. This sequence of testing encouraged the subjects to use the total available range of Likert scores. The first two subjects that did not receive the second anchor in the second trial were regarded as pilot subjects and excluded from the analysis, leaving a total of nine subjects. The subjects were not informed that these first stimuli were anchors. Similar methods deploying such hidden references (anchors) include the Multiple Stimuli with Hidden Reference and Anchor test (International Telecommunication Union 2014).

### Characteristics of the TNR Algorithms: Noise Attenuation and Speech Distortion

**Fig. 3.**
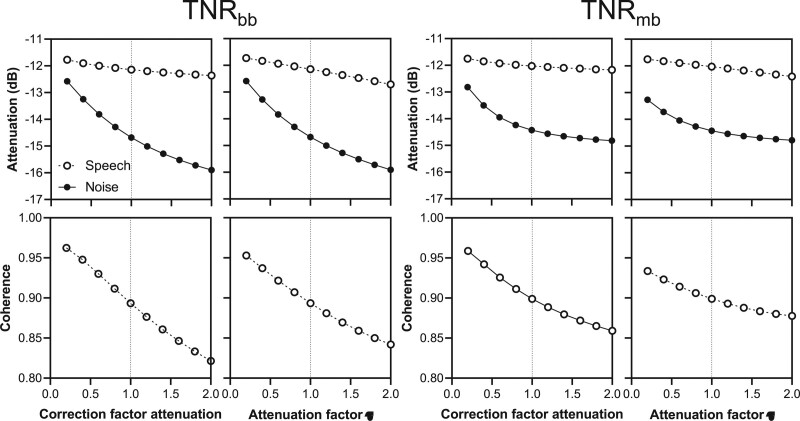
Noise attenuation and speech distortion of a broadband transient noise reduction (TNR_bb_) algorithm and a multiband variant (TNR_mb_) as a function of the two variable settings attenuation and release time (*τ*). Attenuation was defined as the root-mean-square (rms) of the signal. Speech distortion was expressed as the coherence.

Two important outcome measures for the effectiveness of noise reduction strategies in general are the (intended) attenuation of the noise and the (unintended, but inevitable) distortion of the speech signal. To characterize these two measures as a function of attenuation and τ, we calculated the attenuation of noise and the distortion of speech. Attenuation was characterized by calculating the rms value of the original input and of the output after the TNR algorithm was applied. The rms values were calculated by taking the square root of the summed squared samples of the wave files before and after applying the TNR algorithm. The rms values were converted to a dB-scale according to: attenuation = 10·^10^log(rms_output_/rms_input_). The distortion of the speech was characterized by computing the coherence of the speech (Kates & Arehart 2004) by comparing the input speech with the output after applying the TNR algorithm. Coherence is a dimensionless measure for distortion of a signal. It can adopt values between zero and one and indicates how well two signals correlate to each other at a particular frequency. A coherence of 1 means that speech signals before and after filtering are identical. A value of 0 means there is no correlation between the input and output speech (Yousefian & Loizou 2012). Because distortion is expectedly most important for those frequency bands involved in speech understanding, we calculated the coherence on ^1^/_3_ octave bands between 160 Hz and 10 kHz and applied weighting factors to them as reported by Killion & Mueller (2010). The input speech material was generated by concatenating 20 random sentences from the Dutch/Flemish Matrix test corpus into a 40-second speech file. The noise file was 5 seconds and was looped to match the length of the speech material. The resulting data at default settings are presented in Figure 3. It can be seen that both algorithms substantially attenuated the noise transients but concomitantly decreased speech levels as well. In addition, speech was also distorted by both algorithms. TNR_bb_ generally features more aggressive attenuation but also introduces a larger attenuation of speech and more pronounced speech distortion than TNR_mb_. TNR_bb-std_ equals the settings of TNR_bb_ at a scaling factor of attenuation and τ of 1 (vertical dashed lines in Fig. 3).

### Statistical Analysis

To analyze the differences in SRTs between the four conditions (TNR_off_, TNR_bb-std_, TNR_bb_, TNR_mb_), a repeated measures analysis of variance (RM ANOVA) was used. When the assumption of sphericity was violated, the Greenhouse-Geisser correction (GG-cor) for degrees of freedom was applied (Abdi 2010). Normality testing was performed using the Shapiro-Wilk test. Parametric multiple comparisons testing was performed using Tukey’s post hoc test when all conditions were compared or Sidak’s post hoc tests when only selected pairs were compared. Ratings were performed on a Likert scale and tested for significance using a nonparametric Friedman test followed by Dunn’s multiple comparisons testing against the TNR_off_ condition. Dunn’s post hoc test corrects for the inflation of type I errors when multiple comparisons are made within one statistical test (Dunn 1964). Data analysis was performed with GraphPad Prism (version 8.0.1 for Windows; GraphPad Software, La Jolla, CA, USA).

## RESULTS

### Speech Tests With Personalized Settings

**Fig. 4.**
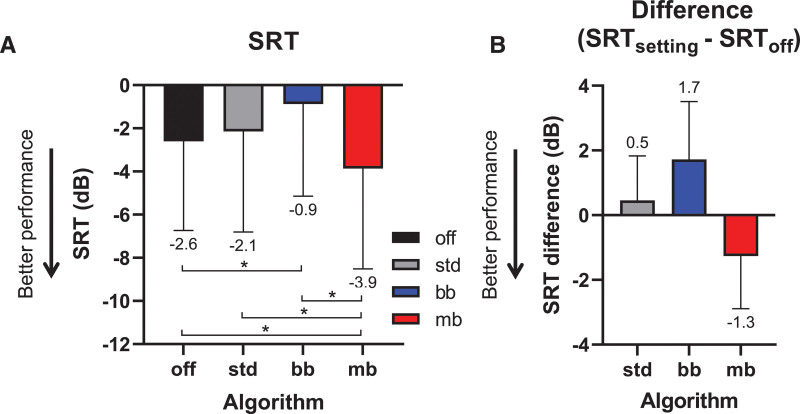
Speech recognition in different conditions (n = 15). A, Speech reception thresholds (SRTs) for four conditions: no transient noise reduction (TNR) (off), clinically available TNR (std), the personalized broadband variant (bb), and the personalized multiband variant (mb). The box indicates the mean, also given below the error bar (lower is better). B, Difference in SRT for TNR_bb-std_, TNR_bb_, and TNR_mb_ relative to TNR_off_. The box indicates the mean, also given below the error bar (lower is better). Error bars: SD; *p < 0.05.

The average SRTs for the four listening conditions are shown in Figure 4A. The figure shows the data obtained without noise reduction (TNR_off_), the clinical standard broadband TNR algorithm (TNR_bb-std_), and the two personalized TNRs, that is, the broadband (TNR_bb_) and multiband (TNR_mb_) algorithms. Normality tests showed that the SRTs were normally distributed for each of the tested TNR algorithms (Shapiro-Wilk test per TNR; *p* > 0.05). RM ANOVA analysis showed a main effect of condition [*F*_(2,30)_ = 12.81; GG-cor *p* < 0.001; *ε* = 0.70]. A Tukey’s multiple comparisons post hoc test revealed that TNR_mb_ with personalized settings significantly improved the SRT (lower SRT) relative to the TNR_off_ condition (mean SRT difference: –1.26 dB; adjusted *p* = 0.04; 95% confidence interval [95% CI] of SRT difference [–0.04 to –2.48]). TNR_mb_ also outperformed TNR_bb-std_ (mean SRT difference: –1.72 dB; adjusted *p* = 0.03; 95% CI of SRT difference [–0.16 to –3.28]) and TNR_bb_ with personalized settings (mean SRT difference: –2.99 dB; adjusted *p* = 0.002; 95% CI of SRT difference [–1.07 to –4.91]). A significant disadvantage was found for TNR_bb_ relative to TNR_off_ (mean SRT difference: +1.73 dB; adjusted *p* = 0.01; 95% CI of SRT difference [0.39 to 3.06]). No significant SRT difference was found between TNR_off_ and the clinically available TNR_bb-std_ (adjusted *p* =0.50). In Figure 4B, the differences in SRT are shown between TNR_off_ and the three settings (TNR_bb-std_, TNR_bb_, TNR_mb_).

To test whether the benefits, or disadvantages, obtained with the TNR algorithms depended on the SNR, and hence on the speech level (noise was always 80 dB SPL equivalent), the SRT differences (SRT_algorithm_–SRT_off_) were correlated with SRT_off_, where SRT_algorithm_ is SRT_bb-std_, SRT_bb_, or SRT_mb_. Because such an analysis correlates two factors that both contain SRT_off_, they are mathematically coupled (Tu & Gilthorpe 2007) and hence correlated a priori. To correct for this coupling (Carlyon et al. 2018), the SRT differences were correlated with the mean of SRT_off_ and SRT_algorithm_. In other words, (SRT_algorithm_–SRT_off_) was correlated with (SRT_off_ + SRT_algorithm_)/2. The SRT differences were not significantly correlated to the SRT for any of the three algorithms (TNR_bb-std_: Pearson’s *r* = 0.39, *p* = 0.15; TNR_bb_: *r* = 0.29, *p* = 0.78; and TNR_mb_: *r* = 0.48; *p* = 0.24).

Five out of 15 subjects explicitly noted that they were not able to recognize the speech that was presented to them during the personalization phase. To test whether this group of subjects differed from the group that was able to recognize some, or all of the words being presented to them, we compared the SRT differences (SRT_algorithm_–SRT_off_) of TNR_bb_ and TNR_mb_ in these two groups with unpaired *t* tests. The group of 10 subjects that were able to recognize the speech performed on average 0.43 dB (95% CI of difference [–1.75 to 2.60]) worse with TNR_bb_, but 1.0 dB better with TNR_mb_ (95% CI of difference [–0.91 to 2.90]). However, these differences were not significantly different (*t*(13) = 0.43, *p* = 0.68 and *t*(13) = 1.12, *p* = 0.28, respectively).

**Fig. 5.**
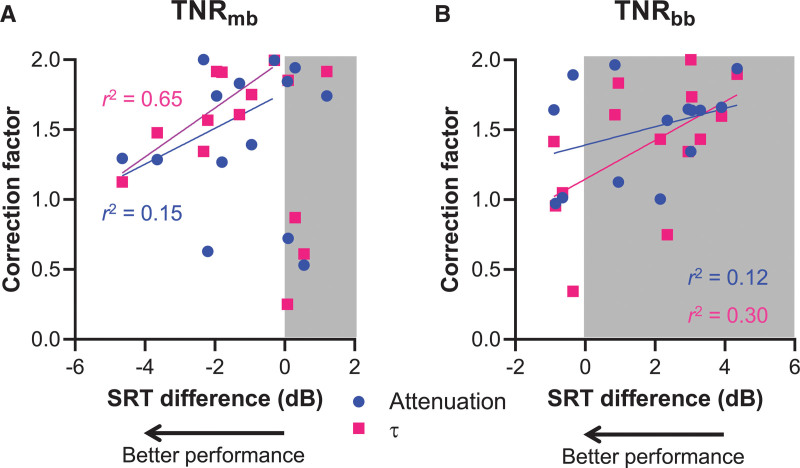
Personalized settings of *τ* and attenuation plotted as a function of the difference in the speech reception threshold (SRT) (n = 14). A, Correlation of the value of the parameter settings attenuation (circles; Pearson’s *r* = 0.39; *p* = 0.31) and *τ* (squares; Pearson’s *r* = 0.81; *p* = 0.009) and the difference in SRT for TNR_mb_ relative to TNR_off_ determined by linear regression. Subjects without benefit (shaded area) were not taken into account. More negative SRT differences mean higher benefit. B, Linear correlation between the value of the parameter settings attenuation (circles; Pearson’s *r* = 0.35; *p* = 0.23) and *τ* (release time; squares; Pearson’s *r* = –0.54; *p* = 0.04) and the difference in SRT for TNR_bb_ relative to TNR_off_. Too little individuals had a benefit to perform a regression analysis on that subpopulation alone. TNR_mb_ indicates multiband transient noise reduction algorithm; TNR_off_, transient noise reduction unfiltered condition.

The individual scaling factors of the personalized TNR_mb_ and TNR_bb_ algorithms are shown in Figure 5 plotted against the SRT difference (SRT_algorithm_–SRT_off_). More negative values of the SRT difference correspond to larger benefits of the algorithm, and more positive values to a bigger disadvantage (grayed-out areas in Fig. 5). Due to technical issues, the personalized settings of S01 were not stored on disc and, hence, only 14 subjects are included in Figure 5. Subject S01 benefited from TNR_mb_ (mean SRT difference across the first two sessions: –2.1 dB), but not from TNR_bb_ (difference: +1.8 dB).

Most subjects (10/15, including S01) benefited from TNR_mb_. By contrast, just 4 out of 15 benefited from TNR_bb_. The lines in the figures represent the best linear fits through the τ and attenuation settings data using linear regression. For TNR_mb_, only those subjects that benefited were included (Fig. 5A), whereas all the data were used to fit the lines of TNR_bb_ (Fig. 5B). For TNR_mb_, it can be seen that most subjects tended to increase the values of τ and attenuation (scaling factors > 1), but that those subjects choosing scaling factors near 1 (i.e., the default values) gained the highest benefits for both settings. Similarly, most subjects chose to increase the scaling factors for TNR_bb_, in the personalization experiment. For this TNR_bb_, however, even small increases in the scaling factors were associated with decreased speech recognition.

### Collapsing the Personalized Data Into an Optimal Group-Based Setting for TNR_mb_

To determine whether the relationship between preferred setting and benefit for TNR_mb_ was causal, we attempted to further optimize the settings of this algorithm by using the regression data shown in Figure 5A in the hope of providing a uniform improvement in SRT. The linear fit analysis included only those subjects that benefited from the algorithm (n = 9) and excluding those subjects that showed a disadvantage (n = 5). An exponential fit was also attempted to account for the apparent leveling off when the disadvantageous settings were taken into account (but not used in the fit). This analysis, however, yielded regressions that did not converge properly for both τ and attenuation. Applying a linear fit, the SRT difference in this subset of subjects showed a significant, positive association with τ (Pearson’s *r* = 0.81; *p* = 0.009), but not with attenuation (Pearson’s *r* = 0.39; *p* = 0.31). Linear interpolation to the most favorable SRT difference observed in the group (S14, –4.7 dB) yielded a group-based attenuation and τ setting, namely a scaling factor of 1.17 and 1.19, respectively. The group-derived values that were actually used in the 3rd session were computed based on interim data (n = 12/15). These values (attenuation: 1.06, τ: 1.12) differed slightly from the final group-based scaling factors of the attenuation and τ determined after the study was finished.

**Fig. 6.**
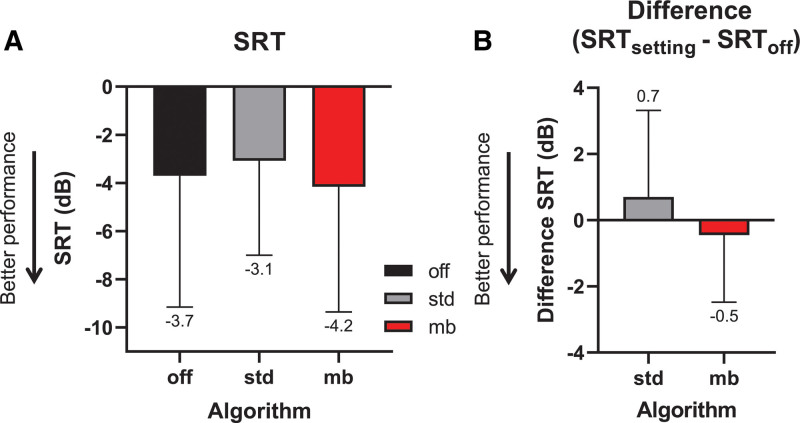
Speech recognition using the group-based settings (n = 15). A, Mean speech reception thresholds (SRTs) for four transient noise reduction (TNR) conditions: no TNR (off), the clinically available standard TNR (std) and the group-based multiband variant (mb). Means are provided numerically below the error bar. B, Same data as in (A), but plotted as the mean difference in SRT for TNR_bb-std_ and TNR_mb_ relative to TNR_off_. Error bars: SD.

The average SRTs for the optimal group-based settings of TNR_mb_ are shown in Figure 6, alongside the data obtained with TNR_off_ and TNR_bb-std_. The RM ANOVA indicated that there was no significant effect of the type of algorithm (TNR_off_, TNR_bb-std_, and TNR_mb_) on the SRT [*F*_(2,14)_ = 1.37; GG-cor *p* = 0.27; *ε* = 0.88]. Figure 6B shows the (statistically not significant) differences between the SRT obtained with TNR_bb-std_ and TNR_mb_ relative to TNR_off_.

### Comparison of the Personalized and Group-Based Settings of TNR_mb_

**Fig. 7.**
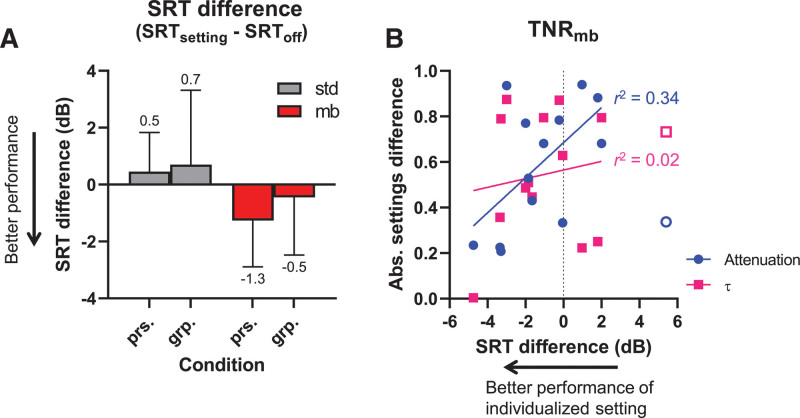
Speech recognition and parameter settings using the group-based settings. A, Mean speech reception threshold (SRT) differences (SRT of the algorithms minus SRT_off_) for the personalized (prs) and group-based (grp). Error bars: SD; n = 15. B, Difference magnitudes of personalized and group-based attenuation (circles) and *τ* (squares) of TNR_mb_ plotted against the SRT differences (SRT with personalized settings minus SRT with group-based settings plotted in [A]; N = 14). Linear regression was performed excluding one subject who showed an exceptionally large disadvantage of TNR_mb_ (open symbols). This analysis yielded a significant correlation for the attenuation setting of TNR_mb_ (Pearson’s *r* = 0.58; *p* = 0.04). TNR_bb-std_ indicates clinical standard broadband transient noise reduction algorithm SoundRelax; TNR_mb_, multiband transient noise reduction algorithm; TNR_off_, transient noise reduction unfiltered condition.

While a significant benefit was observed with TNR_mb_ using personalized settings (Fig. 4A), no such effect was seen using the group-based settings (Fig. 6A). The SRT differences obtained with personalized settings of TNR_mb_ and those obtained with group-based settings are replotted (from Figs. 4B and 6B) side-by-side in Figure 7A, alongside TNR_bb-std_ for reference. An RM ANOVA showed a significant main effect of the TNR [*F*_(1,14)_ = 8.99; GG-cor *p* = 0.045; *ε* = 0.79]. However, a Sidak’s post hoc multiple comparisons test between the personalized and group-based settings showed no significant difference for TNR_mb_ (mean SRT difference: 0.81 dB; adjusted *p* = 0.43; 95% CI of SRT difference [–2.49 to 0.86]). The settings of TNR_bb-std_ were identical between the first two sessions and the 3rd, and TNR_bb-std_ was therefore analyzed as a reference only. As expected, no significant difference was found for TNR_bb-std_ (SRT difference: 0.25 dB; adjusted *p* = 0.94; 95% CI of SRT difference [–2.19 to 1.70]).

Although the post hoc test did not reveal a significant difference between personalized and group-based settings (Fig. 6A), TNR_mb_ with personalized settings resulted in a significant improvement over TNR_off_ and TNR_bb-std_ (Fig. 4A). To better understand the association between the results from the personalized settings and the group settings, we examined whether there was a correlation between the magnitude of the difference between the personalized and group-based attenuation and τ and the SRT difference obtained with TNR_mb_ (Fig. 7B). This SRT difference was calculated by subtracting the SRT using the group-based settings from the personalized settings. Negative SRT differences in Figure 7B hence indicate a benefit of the personalized over the group-based settings, positive values a disadvantage. Under the assumption that personalized settings are preferable, this analysis hence could reveal whether a larger deviation from the personalized setting resulted in smaller benefits of TNR_mb_. Linear regression showed a trend toward SRTs growing worse when the magnitude of the difference between the personalized and group-based settings was larger. Excluding the one subject with a gross deterioration of the SRT when using the personalized setting (S15, open symbols), the trend was significant for the attenuation setting (Pearson’s *r* = 0.58, *F*(1,11) = 5.64, *p* = 0.04), but not for τ (*r* = 0.14; *F*(1,11) = 0.23, *p* = 0.64).

### Subjective Ratings

**Fig. 8.**
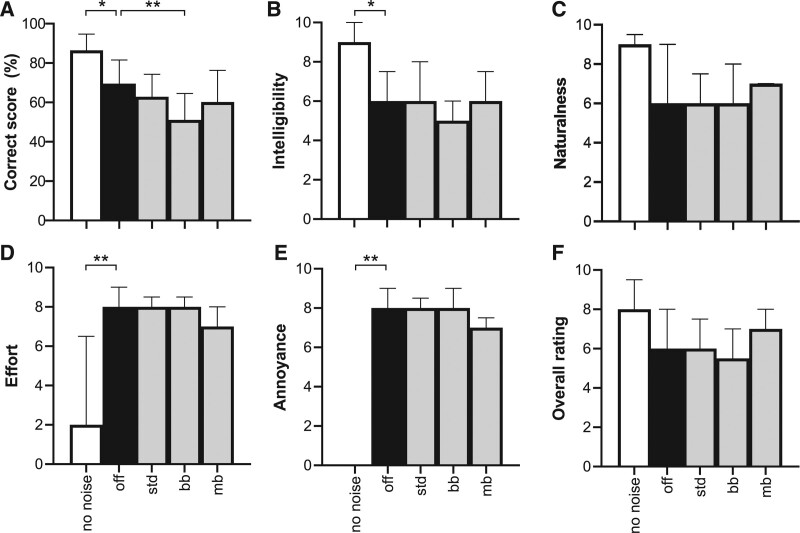
Results of the rating experiment (n = 9). A, Fraction of correctly identified words, (B) intelligibility, (C) naturalness, (D) listening effort, (E) annoyance, and (F) overall rating. No significant differences between transient noise reduction (TNR) algorithms and control (off) were found. Bars represent the mean with SD (A) or median with interquartile range (B–F). bb indicates personalized broadband transient noise reduction; mb, personalized multiband transient noise reduction; std, standard broadband transient noise reduction.

The results of the subjective ratings, along with the word recognition scores, are shown in Figure 8. In the figure, the first value of each graph (white bar) represents the listening condition without noise and without any TNR algorithm applied (i.e., the first anchor). The second value (black bar) represents the listening condition with impulse noise but without the application of a TNR algorithm (TNR_off_, the second anchor). The following gray bars represent TNR_bb-std_, and the personalized TNR_bb_ and TNR_mb_. The group-based settings for TNR_mb_ were excluded from this test. Statistical significance testing was performed using a nonparametric Friedman test on the rating data, and a parametric RM ANOVA on the correct scores. All of the tests showed a main effect when the no-noise condition was included (*p* < 0.01). However, a multiple comparisons Dunn’s post hoc test with TNR_off_ as reference condition (black bar) showed that none of the algorithms, including TNR_mb_ with personalized settings, significantly differed from TNR_off_ in any of the rating tests. The post hoc test on the correct scores revealed that TNR_bb_ was the only algorithm that significantly affected the correct score; it decreased the score by 18% relative to TNR_off_ (adjusted *p* = 0.01). The observed main effects of listening conditions appeared to be caused mainly by the difference between TNR_off_ and the no-noise condition, as it was significant in all conditions (*p* < 0.05), except for the naturalness and overall rating, where no post hoc differences were found at all.

## DISCUSSION

In this study, we demonstrated that personalizing the settings of TNR_mb_ significantly improves SRTs by 1.3 dB in transient noise relative to TNR_off_. By contrast, personalization of the settings of TNR_bb_ had a negative impact on the SRT 1.7 dB. TNR_bb-std_ did not affect the SRT. Group-based settings eliminated the beneficial effect of TNR_mb_ and a correlation analysis showed that larger differences between personalized and group settings of the attenuation of TNR_mb_ significantly increased SRTs (i.e., worse speech recognition). Subjective ratings were not significantly affected by any of the algorithms tested. When collapsing the personalized settings to determine the optimal group-averaged setting, the beneficial effect of TNR_mb_ was abolished. A larger deviation from the personalized attenuation setting resulted in a significant decline in performance of TNR_mb_. This finding supports the conclusion that personalizing TNR_mb_ settings can benefit speech recognition, at least for the attenuation setting. Strictly speaking, a correction for multiple comparisons testing should have been performed for this analysis, since the correlation of both attenuation and τ were compared. After a Bonferroni correction, the correlation of τ was not significant anymore (corrected *p* = 0.074). Further study of the underlying reasons for the benefits of parameter personalization of the TNR_mb_ is hence warranted to obtain more conclusive evidence. Because the multiband nature of the algorithm seems to be the decisive component in its beneficial effects, it seems worth investigating how much benefit can be achieved by personalizing the fitting of TNR_mb_ for each individual frequency band. Given current clinical fitting practice, any hypothetical additional benefit would have to be carefully weighed against the increased burden on the audiologist, but as fitting workflows gradually progress toward allowing subjects to fine-tune certain aspects of their fitting themselves, this trade-off may shift in favor of higher degrees of personalization over time without overburdening clinicians.

The personalized settings of both TNR_mb_ and TNR_bb_ were characterized by scaling factors that were predominantly larger than 1. In other words, most subjects tended to increase both the release time τ and attenuation factors favoring more aggressive filtering. It is interesting that both parameter settings showed a trend toward worse speech recognition with higher scaling factors for both algorithms, possibly because of progressively larger speech distortion at more aggressive parameter settings (Fig. 3). Collapsing the data and extrapolation to the largest benefit indeed resulted in scaling factors closer to 1, that is, approaching the default settings (Fig. 5). However, this procedure abolished the beneficial group effects of TNR_mb_ altogether (Fig. 6). This finding shows that imposing settings chosen by subjects experiencing the most benefit on those with less benefit does not necessarily improve performance, and doing so in fact deteriorated group performance. These findings support the use of personalized settings to fit TNR_mb_, rather than a one-size-fits-all approach. We note that the linear relation was statistically significant only for the τ setting of TNR_mb_ and not for the attenuation parameter (Fig. 5) and more research on the dependence of personalized settings and speech recognition with TNR algorithms is necessary to provide more definitive answers. Until then, the present results point toward TNR_mb_ with personalized parameter settings to be the only beneficial algorithm under the conditions tested.

A limitation of our personalization method was that the first of three iterations confined the parameter space of the subsequent iterations. While halving of the available parameter space can potentially improve the accuracy of the preferred scaling factor estimation, it may have resulted in suboptimal scaling factors for those subjects that failed to explore the full parameter space in the first iteration.

TNR_mb_ has been studied before in a study from Dyballa et al. (2016), where it was tested with fixed attenuation and τ and compared with the clinical standard TNR_bb-std_, also with fixed parameter settings. The algorithms were tested in different noise conditions, one of which was the same cafeteria noise as used in the present study. In cafeteria noise, the multiband algorithm improved speech intelligibility significantly with 2.9 dB and 2.4 dB relative to the TNR_bb-std_ and the condition without the algorithm, respectively. We have also found significant, albeit smaller differences of 1.8 and 1.3 dB, respectively, when using personalized settings. With group-based settings, we did not find a significant effect at all. One potential explanation for the lesser benefits found in the present study is that the τ and attenuation settings were set at default in the study of Dyballa et al. (2016), that is, corresponding to a scaling factor of 1 in this study. Another possible cause for the different outcomes is that their population consisted of better performing subjects. In their article, no CVC scores are provided, making it impossible to compare our subjects based on the speech-recognition performance in quiet. However, only five of the 15 subjects in our study had an SRT equal to or better than the SRT of their worst performing subject in the condition without TNR. The mean speech level of our subjects was 77 dB, while the median in their subject group was 72 dB using the same noise level of 80 dB. In other words, their subjects were doing the test at an SNR that was 5 dB worse, on average. This might have affected the TNR effectivity, as the detection of transients is expectedly more efficient at lower SNRs, because the speech signal is smaller and transients are thus more pronounced and more efficiently detected and attenuated (Dyballa et al. 2016). This same argument may explain the unexpected finding that TNR_mb_, in contrast to its beneficial effect on the SRT, did not affect word correct scores obtained at a more favorable speech level of SRT + 3 dB.

It is of interest to investigate whether the performance of TNR_mb_ and other related algorithms depends on the SNR used to test them. We investigated this post hoc by correlating SRT differences obtained with the TNR algorithms to each individual’s SRT, which proved not statistically significant. A more systematic approach can be realized by varying SNRs within subjects and test the performance of the TNR algorithms. This would allow for a more robust, repeated-measures analysis. Until more conclusive evidence is obtained, care should be taken to extrapolate our findings to the CI population at large. The mean CVC phoneme score in quiet of 91% (equivalent to a word score of 81%) in our subject population can be regarded as above-average in our clinic. Lesser performing individuals may require higher SNRs and may hypothetically benefit less from TNR_mb_.

The clinically available broadband algorithm was studied previously by Dingemanse et al. (2018). They reported no significant effect of the algorithm on speech intelligibility using kitchen sounds (clattering dishes) as background noise. This is in line with our study, since we did not find a significant effect with this algorithm in cafeteria noise with clattering dishes and multi-talker babble noise. In contrast, Dyballa et al. (2015) reported a significant effect of 0.4 dB with a broadband algorithm with strong noise reduction settings in the same cafeteria noise. They found a larger improvement in speech intelligibility in hammering noise (1.7 dB for stronger noise reduction settings, 0.6 dB with weaker settings). This can, possibly, be explained by the artificial nature of the hammering signal, which provided near-optimal conditions for the detection mechanism. All the noise transients had the same rapid onset and occurred at high enough rates to affect speech recognition, while at the same time, the hammering frequency was sufficiently low that the transients could be recognized as separate events. The cafeteria noise used in the present study consisted of clattering dishes mixed with background babble noise. This type of noise is more realistic and varied in terms of onset and decay times, peak amplitude, spectral content, and event rate (see Dyballa et al. [2015] for a detailed analysis of the cafeteria noise).

In the present study, personalized TNR_bb_ settings proved to worsen speech recognition in most subjects relative to TNR_off_. We do not think the personalization failed in this case. Instead, it is known that users may subjectively prefer TNR algorithms, even though they negatively impact speech recognition (Dingemanse et al. 2018). Hence, we expect that the subjects chose those settings that subjectively sounded best to them.

In terms of the subjectively perceived effects, none of the differences between the various tested algorithms and TNR_off_ were significant in the present study. In the study of Dyballa et al. (2016), subjective quality tests revealed that TNR_mb_ significantly improved listening comfort and speech clarity ratings in cafeteria noise. The overall preference was not significantly better. The fact that they were able to find significant effects, while we were not, was likely caused by procedural differences. In their study, subjects were equipped with a touch screen that allowed playing and replaying the material in any order and for any number of repeats. These side-by-side comparisons are cognitively less demanding than the presentation of serial blocks of ten sentences per listening condition, as deployed in this study. In the latter case, each subjective sound quality needs to be remembered and compared with the previous conditions. Further, while we anchored the data by presenting the ideal condition (no noise) and hardest condition (noise, without TNR algorithm) first in most subjects, it may be preferable to allow the subject to relisten to the anchors and TNR stimuli at will to eliminate any effects of the stimulus-presentation order. For instance, the Multiple Stimuli with Hidden Reference and Anchor test deploys side-by-side comparisons, including hidden anchors (International Telecommunication Union 2014).

The single-masked study design may have resulted in bias, since the experimenter was aware of the listening condition being tested at any given time. This may have resulted in bias during the speech recognition tests, because the subjects’ responses were manually scored by the experimenter. It may also have influenced the subjective tests, given that verbal interaction was possible between the experimenter and the test subject.

Another limitation of the present study is the method we used to obtain the individual noise reduction parameters. Five out of 15 subjects were not able to understand the speech properly, which indicates that the task was too challenging. A more favorable SNR could have been established by lowering the noise level (set at 80 dB here). We designed the task based on the study of Dyballa et al. (2015), including the type of noise and the SNR. However, we did not foresee that the speech was unintelligible for so many subjects. As a result, one third of the subjects based their preferred settings solely on the sound quality, rather than the intended speech intelligibility. This may have affected their choice of settings choice that did not necessarily improve speech recognition. The SRT differences of the two groups of subjects that recognized the speech (n = 10) and those that did not (n = 5) did not statistically differ for TNR_bb_ nor TNR_mb_. However, the unpaired nature and small sample sizes substantially reduce the power of such a test. In addition, we did not systematically register whether the subjects could recognize the speech, or the extent to which they recognized the text. Therefore our statistical results are indicative only. Future studies can negate the issue of speech intelligibility altogether by using personalized SNRs for the personalization procedure, for example, based on the subject’s own SRT. The potential lack of statistical power due to the relatively small study population included is another limitation of this study. We corrected all post hoc tests of the individual ANOVA analyses for multiple comparisons. However, we performed multiple such ANOVA tests, and strictly spoken, we should have taken this into account as well, to account for inflated type I errors, for example with a Bonferroni correction. We did not perform such a correction, because it would further reduce statistical power of this study. Furthermore, we did not obtain a retest of the speech tests with group-based settings, which may have resulted in higher variability and, therefore, lower statistical power. The lack of power could be an explanation of the fact that the difference between the personalized and group-based settings was not significant, despite a trend being visible for TNR_mb_ (Fig. 7). In addition, no retest of the personalization of the attenuation and τ settings was obtained, which expectedly would have yielded more reliable estimates.

In the present study, both the TNR algorithms and dual-loop AGC were simulated. As a result, the results described in this study reflect the joint effect of the TNR algorithms and the AGC acting in series. While this complicates the interpretation of the present results, removing the AGC from the signal path would have resulted in a setup far removed from any clinically relevant fitting and would consequently offer very little practically relevant insight regarding the effect of TNR on patient outcomes. Nonetheless, interactions between TNR algorithms and the AGC warrants further investigation. Driving the AGC into strong compression or saturation with a high-intensity transient will affect the dynamics of the system for some period of time after the offset of the transient. The potential benefit of any TNR algorithm as a preprocessing step to the AGC critically depends on two characteristics: whether the algorithm is faster and more reliable in suppressing an undesired transient noise and whether its attenuation response following the transient offset subsides faster than the response of the AGC to the nonattenuated transient. Optimization of the interplay between the currently tested algorithms and the AGC can be a promising area of future research.

## CONCLUSIONS

The present study shows that the personalization of a multiband TNR algorithm improves speech recognition around the SRT in CI users. A broadband TNR algorithm, either with fixed or personalized settings, does not enhance speech recognition in transient cafeteria noise. Future research is required to investigate the underlying reason for the benefit of personalized noise reduction settings, and the lack of it at more favorable SNRs. The current clinical TNR algorithm applied in Advanced Bionics’ speech processors (SoundRelax) is a broadband filter that does not support personalization of its settings. Our results suggest that future iterations of the algorithm can benefit from multiband, instead of broadband processing, and from adding an option to personalize its parameter settings during fitting. Ideally, the functionality of the currently used MATLAB script to personalize the settings should be included in the clinical fitting software (SoundWave in case of Advanced Bionics devices), including the option to enter customized values for the release time τ and attenuation factor. The development of fitting workflows that allow CI users to fine-tune certain aspects of their fitting themselves can further facilitate the personalization of noise reduction algorithms.

## ACKNOWLEDGMENTS

The authors thank the subjects for their time and dedication and Raphael Koning (Advanced Bionics, Hannover, Germany) for technical assistance.

## References

[R1] Abdi H. (Salkind N. (Ed.), The Greenhouse-Geisser correction. In Encyclopedia of Research Design, vol. 2010). 1 (pp. Sage.545–548).

[R2] Boyle P. J. Büchner A. Stone M. A. Lenarz T. Moore B. C. (Comparison of dual-time-constant and fast-acting automatic gain control (AGC) systems in cochlear implants. Int J Audiol, 2009). 48, 211–221. 19363722 10.1080/14992020802581982

[R3] Brand T. Kollmeier B. (Efficient adaptive procedures for threshold and concurrent slope estimates for psychophysics and speech intelligibility tests. J Acoust Soc Am, 2002). 111, 2801–2810. 12083215 10.1121/1.1479152

[R5] Carlyon R. P. Cosentino S. Deeks J. M. Parkinson W. Arenberg J. G. (Effect of stimulus polarity on detection thresholds in cochlear implant users: Relationships with average threshold, gap detection, and rate discrimination. J Assoc Res Otolaryngol, 2018). 19, 559–567. 29881937 10.1007/s10162-018-0677-5 PMC6226408

[R6] DiGiovanni J. J. Davlin E. A. Nagaraj N. K. (Effects of transient noise reduction algorithms on speech intelligibility and ratings of hearing aid users. Am J Audiol, 2011). 20, 140–150. 21940982 10.1044/1059-0889(2011/10-0007)

[R7] Dingemanse J. G. Vroegop J. L. Goedegebure A. (Effects of a transient noise reduction algorithm on speech intelligibility in noise, noise tolerance and perceived annoyance in cochlear implant users. Int J Audiol, 2018). 57, 360–369. 29334269 10.1080/14992027.2018.1425004

[R8] Dunn O. J. (Multiple comparisons using rank sums. Technometrics, 1964). 6, 241–252.

[R9] Dyballa K. H. Hehrmann P. Hamacher V. Lenarz T. Buechner A. (Transient noise reduction in cochlear implant users: A multi-band approach. Audiol Res, 2016). 6, 154. 27942372 10.4081/audiores.2016.154 PMC5134678

[R10] Dyballa K. H. Hehrmann P. Hamacher V. Nogueira W. Lenarz T. Büchner A. (Evaluation of a transient noise reduction algorithm in cochlear implant users. Audiol Res, 2015). 5, 116. 26779325 10.4081/audiores.2015.116 PMC4698598

[R11] Firszt J. B. Holden L. K. Reeder R. M. Skinner M. W. (Speech recognition in cochlear implant recipients: Comparison of standard HiRes and HiRes 120 sound processing. Otol Neurotol, 2009). 30, 146–152. 19106769 10.1097/MAO.0b013e3181924ff8 PMC3603702

[R12] Fredelake S. Geissler G. Buchner A.. (Die adaptive kategoriale Lautheitsskalierung mit direkter elektrischer Stimulation beim Cochlea-Implantat. 2014). In 17. Jahrestagung der Deutschen Gesellschaft für Audiologie; March 13, 2014; Oldenburg, Germany.

[R13] Henderson D. Hamernik R. P. (Impulse noise: Critical review. J Acoust Soc Am, 1986). 80, 569–584. 3745686 10.1121/1.394052

[R14] Hernandez A. R. Chalupper J. Powers T. A. (An assessment of everyday noises and their annoyance. Hear Rev, 2006). 13, 16–20.

[R15] International Telecommunication Union. (Recommendation ITUR BS.1534-2 – Method for the subjective assessment of intermediate quality level of audio systems. 2014). BS Series, Broadcasting service (sound). https://www.itu.int/rec/R-REC-BS.1534-3-201510-I/en

[R16] Kates J. Arehart K. (Coherence and the speech intelligibility index. J Acoust Soc Am, 2004). 115, 2604–2604. 10.1121/1.1862575 15898663

[R17] Keidser G. O’Brien A. Latzel M. Convery E. (Evaluation of a noise-reduction algorithm that targets non-speech transient sounds. Hear J, 2007). 60, 29–39.

[R18] Keshavarzi M. Baer T. Moore B. C. J. (Evaluation of a multi-channel algorithm for reducing transient sounds. Int J Audiol, 2018). 57, 624–631. 29764254 10.1080/14992027.2018.1470336

[R19] Killion M. C. Mueller H. G. (Twenty years later: A NEW Count-The-Dots method. Hear J, 2010). 63, 10–17.

[R20] Kokkinakis K. Hazrati O. Loizou P. C. (A channel-selection criterion for suppressing reverberation in cochlear implants. J Acoust Soc Am, 2011). 129, 3221–3232. 21568424 10.1121/1.3559683 PMC3108395

[R21] Kollmeier B. Warzybok A. Hochmuth S. Zokoll M. A. Uslar V. Brand T. Wagener K. C. (The multilingual matrix test: Principles, applications, and comparison across languages: A review. Int J Audiol, 2015). 54(Suppl 2), 3–16. 26383182 10.3109/14992027.2015.1020971

[R22] Levitt H. (Transformed up-down methods in psychoacoustics. J Acoust Soc Am, 1971). 49, 467–477. 5541744

[R23] Liu H. Zhang H. Bentler R. A. Han D. Zhang L. (Evaluation of a transient noise reduction strategy for hearing AIDS. J Am Acad Audiol, 2012). 23, 606–615. 22967735 10.3766/jaaa.23.8.4

[R24] Moore B. C. Glasberg B. R. (A comparison of four methods of implementing automatic gain control (AGC) in hearing aids. Br J Audiol, 1988). 22, 93–104. 3390637 10.3109/03005368809077803

[R25] Nelson P. B. Jin S. H. Carney A. E. Nelson D. A. (Understanding speech in modulated interference: Cochlear implant users and normal-hearing listeners. J Acoust Soc Am, 2003). 113, 961–968. 12597189 10.1121/1.1531983

[R26] Tu Y. K. Gilthorpe M. S. (Revisiting the relation between change and initial value: A review and evaluation. Stat Med, 2007). 26, 443–457. 16526009 10.1002/sim.2538

[R28] World Medical Association. (World medical association declaration of helsinki: Ethical principles for medical research involving human subjects. JAMA, 2013). 310, 2191–2194. 24141714 10.1001/jama.2013.281053

[R29] Yousefian N. Loizou P. C. (A dual-microphone speech enhancement algorithm based on the coherence function. IEEE Trans Audio Speech Lang Process, 2011). 20, 599–609. 22207823 10.1109/TASL.2011.2162406 PMC3246289

[R30] Zeng F. G. Nie K. Stickney G. S. Kong Y. Y. Vongphoe M. Bhargave A. Wei C. Cao K. (Speech recognition with amplitude and frequency modulations. Proc Natl Acad Sci U S A, 2005). 102, 2293–2298. 15677723 10.1073/pnas.0406460102 PMC546014

